# The Microstructure and Thermal Conductive Behavior of Three-Dimensional Carbon/Carbon Composites with Ultrahigh Thermal Conductivity

**DOI:** 10.3390/ma17050983

**Published:** 2024-02-20

**Authors:** Baoliu Li, Chenyu Zhu, Huitao Xu, Yudan Qin, Changchun Shan, Fang Gao, Jianguang Guo, Zhijun Dong, Xuanke Li

**Affiliations:** 1 Hubei Province Key Laboratory of Coal Conversion and New Carbon Materials, Wuhan University of Science and Technology, Wuhan 430081, China; zhuchenyu@wust.edu.cn (C.Z.); qinyudan@wust.edu.cn (Y.Q.); gaofang0415@wust.edu.cn (F.G.); guojianguang@wust.edu.cn (J.G.); dongzhijun@wust.edu.cn (Z.D.); 2 Hubei Province Pilot Base on Coal Conversion and New Carbon Materials, Wuhan University of Science and Technology, Wuhan 430081, China; 3 Baowu Carbon Technology Co., Ltd., Shanghai 201999, China; huitaoxu1995@163.com (H.X.); shanchangchun@baosteel.com (C.S.)

**Keywords:** mesophase pitch-based carbon fibers, PAN-based carbon fibers, hot pressing, impregnation, high thermal conductivity

## Abstract

Carbon-based composite materials, denoted as C/C composites and possessing high thermal conductivity, were synthesized utilizing a three-dimensional (3D) preform methodology. This involved the orthogonal weaving of mesophase pitch-based fibers in an X (Y) direction derived from low-temperature carbonization, and commercial PAN-based carbon fibers in a Z direction. The 3D preforms were saturated with mesophase pitch in their raw state through a hot-pressing process, which was executed under relatively low pressure at a predetermined temperature. Further densification was achieved by successive stages of mesophase pitch impregnation (MPI), followed by impregnation with coal pitch under high pressure (IPI). The microstructure and thermal conductivity of the C/C composites were systematically examined using a suite of analytical techniques, including Scanning Electron Microscopy (SEM), X-ray Diffraction (XRD), and PLM, amongst others. The findings suggest that the volumetric fraction of fibers and the directional alignment of the mesophase pitch molecules can be enhanced via hot pressing. The high graphitization degree of the mesophase pitch matrix results in an increased microcrystalline size and thus improved thermal conductivity of the C/C composite. Conversely, the orientation of the medium-temperature coal pitch matrix is relatively low, which compensates for the structural inadequacies of the composite material, albeit contributing minimally to the thermal conductivity of the resultant C/C composites. Following several stages of impregnation with mesophase pitch and subsequent impregnation with medium-temperature coal pitch, the 3D C/C composites yielded a density of 1.83 and 2.02 g/cm^3^. The thermal conductivity in the X (Y) direction was found to be 358 and 400 W/(m·K), respectively.

## 1. Introduction

Hypersonic technology has emerged as a critical area of interest within the aerospace sector in the 21st century, exhibiting a substantial potential for dual-use applications [1,2,3]. However, vehicles such as space shuttles returning to the atmosphere and hypersonic aircraft operating within the atmospheric realm, undergo prolonged periods of service (exceeding 1000 s), characterized by low density and substantial heat flux magnitudes (MW/m^2^), resulting in continuous heating. The accumulated heat is significant, leading to surface temperatures exceeding 2000 °C in certain specialized components such as the nose cone, the leading edge of sharp wings, and the hot end parts of the scramjet [4,5,6,7]. High thermal conductivity carbon/carbon (C/C) composites [8,9] reinforced with mesophase pitch-based carbon fibers [10,11,12,13] are considered optimal candidate materials due to their exceptional characteristics. These include high thermal conductivity, resistance to high temperatures, high ablative resistance, low density, low expansion coefficient, and excellent high-temperature strength. These properties enable the rapid transfer of heat from high heat flow regions to lower heat flow regions, subsequently reducing the surface temperature and local thermal stress in the high heat flow areas [3]. Consequently, this simplifies the design of heat protection components and enhances the reliability of these elements.

Presently, the majority of research studies on high thermal conductivity C/C composites focus predominantly on unidirectional (1D) structures, with relatively fewer investigations into two-dimensional (2D) and three-dimensional (3D) high thermal conductivity C/C composites. Notably, Klett et al. [14] utilized T300, P55, and high thermal conductivity strip pitch-based carbon fibers, along with AR mesophase pitch powder, as foundational materials. By implementing a methodology of hot pressing, oxidation, carbonization, and graphitization, a unidirectional prepreg tow was produced that was coated in mesophase pitch powder, resulting in three distinct types of 1D C/C composite materials. The thermal conductivity at room temperature, measured along the fiber length, was found to be 80.5 W/(m·K), 135.5 W/(m·K), and 214 W/(m·K), respectively. In a similar vein, Amoco [15] used pre-oxidized mesophase pitch-based fibers as raw materials, creating unbonded self-reinforced 1D C/C composites via hot pressing, carbonization, and graphitization. It was observed that when the volume content of the carbon fiber was 82%, the C/C composites, after undergoing graphitization at 3000 °C, exhibited a thermal conductivity of 746 W/(m·K) at room temperature along the fiber length. Ma Zhaokun et al. [16,17] prepared 1D C/C composites using circular and strip pre-oxidized mesophase pitch-based fibers as raw materials. These were processed through a hot press–graphitization process. The resulting axial thermal conductivity of C/C composites prepared with ribbon fibers and circular fibers were 837 W/(m·K) and 649 W/(m·K), respectively. Interestingly, the thermal conductivity of circular fibers was significantly lower compared to the C/C composites prepared with ribbon fibers. Furthering these studies, Yuan Guanming et al. [18] prepared 1D block C/C composites by hot pressing, carbonization, and graphitization. This was achieved using circular pre-oxidized mesophase pitch-base fibers with a diameter of approximately 50 μm and large-sized ribbon fibers with a cross-section width of about 2 mm, and a thickness of about 10 μm. These were used as reinforcement, with mesophase pitch serving as the binder. The room-temperature thermal conductivity along the fiber direction was found to be between 650~734 W/(m·K) and 896 W/(m·K). Huang Dong et al. [19] utilized mesophase pitch-based carbon fiber cloth and mesophase pitch powder as raw materials. They prepared 2D high-thermal carbon composite materials with a fiber volume content of 60% and a density of 2.02 g/cm^3^ through hot pressing and a pitch impregnation–graphitization process. The room-temperature thermal conductivity in the X(Y) direction and Z direction was 429 W/(m·K) and 69 W/(m·K), respectively. Finally, the author [20] employed laboratory-prepared mesophase pitch-based carbon fibers (X and Y direction) and commercial PAN-based carbon fiber (Z direction) to create a preform. This was densified through multiple CVIs and a furan resin impregnation–carbonization–graphitization process. As a result, the 3D C/C composites had a room-temperature thermal conductivity of 235 W/(m·K) in the X(Y) direction and 42 W/(m·K) in the Z direction.

In conclusion, C/C composites with 1D and 2D structures exhibit high thermal conductivity when measured along the length of the fiber. However, their applicability is restricted due to pronounced anisotropy and insufficient interlayer shear force. C/C composites with 3D structures have low thermal conductivity due to low compactness. This study focused on a new method for the rapid preparation of 3D carbon composites with high density and high thermal conductivity. A combination of laboratory-prepared mesophase pitch-based carbon fibers (oriented in the X and Y directions) and commercial PAN-based carbon fibers (oriented in the Z direction) were utilized to construct 3D preform materials. The initial step was to elevate the fiber volume content of the composite through mesophase pitch impregnation, hot pressing, and carbonization. Subsequently, high density 3D C/C composites with high thermal conductivity were synthesized via multiple cycles of mesophase pitch impregnation and medium-temperature coal pitch impregnation, followed by carbonization and graphitization. This research also delved into the impact of the matrix carbon structure and orientation on the structure and properties of C/C composites during the densification process, especially the hot-pressing process. Moreover, the thermal conductivity mechanism of C/C composites was elucidated by integrating the thermal conductivity analyses of these composites.

## 2. Experiment

### 2.1. Preparation of 3D C/C Composites Preform

**Figure 1:**
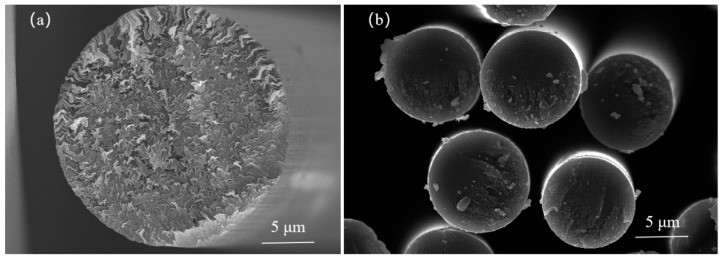
SEM images of carbon fibers: (**a**) mesophase pitch-based carbon fibers, (**b**) PAN-based carbon fibers.

The preform for the 3D C/C composites was constructed as a 3D-woven fabric, structured in three orthogonal dimensions. The X and Y directions were composed of 0.5 K mesophase pitch-based carbon fibers (MPCF), laboratory developed and subsequently carbonized at 500 °C to attain orthogonal arrangement at specified levels. The Z direction utilized HTS-E13-3K PAN-based carbon fibers (PAN-CF) (Teijin Limited, Tokyo, Japan) to accomplish bilateral perforation. Figure 1 displays the SEM images of the mesophase pitch-based carbon fibers and PAN-based carbon fibers. The preform’s bulk density was approximately 0.92 g/cm^3^, with volume fractions of fibers along the X, Y, and Z directions estimated at 23%, 23%, and 4%, respectively. The corresponding properties of MPCF and PAN-CF are detailed in Table 1.

### 2.2. Preparation of 3D C/C Composites

**Figure 2:**
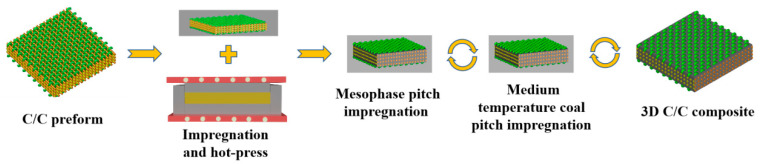
Preparation technology of carbon composite material with high thermal conductivity.

The 3D C/C preform was subjected to mesophase pitch impregnation (MPI) and subsequently densified via 2 MPa hot pressing and carbonization. The prefabricated body was compressed by 20% during molding in the thickness direction, consequently increasing the fiber volume fraction in the composite material by 20%. The 3D C/C composite material was further densified through multiple cycles of mesophase pitch impregnation, followed by carbonization–graphitization, achieving an average density of 1.83 g/cm^3^, and designated as C/C-MPI. Further densification was accomplished by infiltrating the C/C composites with medium-temperature coal pitch via high-pressure impregnation (IPI) and carbonization–graphitization conducted multiple times. Ultimately, 3D C/C composites with an average density of 2.02 g/cm^3^ were produced, referred to as C/C-MPI-IPI. The fabrication methodology for the high thermal conductivity carbon composite material is illustrated in Figure 2.

### 2.3. Detection and Analysis

The thermal conductivity of the C/C composites was assessed using an indirect method. The specimen was segmented into blocks measuring 10 mm × 10 mm × (3–4) mm, and the thermal diffusivity of the material was ascertained using a laser-flash diffusivity apparatus (LFA 457, NETZSCH, Selb, Germany) under ambient temperature conditions. The thermal conductivity of the material was computed by applying the formula λ = α × ρ × C_p_, where α represents the thermal diffusivity, ρ denotes the volume density, and C_p_ signifies the specific heat capacity. The value of Cp for the sample at room temperature was approximately 0.75 J/g·K, which falls within the reported range of 0.72–0.99 J/g·K, as inferred from references [19]. The thermal diffusivity of the materials was gauged using the LFA457Nanoflash TM laser thermal conductivity instrument from NETZSCH Instruments (Selb, Germany).

The optical texture, microstructure, and morphology of the C/C composites were examined using a polarizing light microscope (PLM) (AX10, Carl Zeiss, Jena, Germany) and a field-emission scanning electron microscope (SEM) (FEI Company, Hillsboro, OR, USA). The C/C composites were subjected to X-ray diffraction (XRD) (Philip X’Pert MPD Pro, PANalytical, Almelo, The Netherlands) utilizing Cu Kα radiation (λ = 0.15406 nm) at an accelerating voltage and current of 40 kV and 30 mA, respectively. The interplanar spacing (d_002_) of graphite was calculated using Bragg’s law. Raman experiments of the C/C composites were performed on a Renishaw InVia 2000 Raman microscope (New Mills, UK) using Ar + 514.5 nm radiation for excitation at a power of 25 mW.

## 3. Results and Discussion

### 3.1. Volume Density of 3D C/C Composites

**Figure 3:**
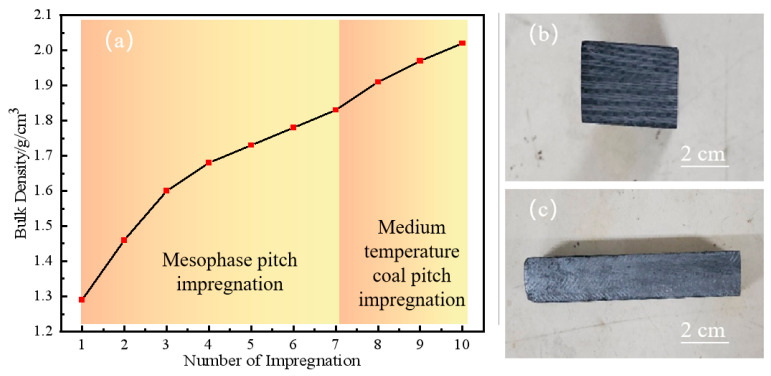
Density curve of 3D C/C composite material with impregnation times (**a**) and physical photos of 3D C/C composite material (**b**,**c**).

Figure 3 delineates the density of 3D C/C composites across various processing stages. As per the figure, the density of the C/C composite materials swiftly escalated to 1.30 g/cm^3^ via mesophase pitch impregnation and hot pressing–carbonization. The density of the C/C composites exhibited a positive correlation with the number of impregnation cycles; however, the rate of increase progressively diminished, with the peak density reaching 1.83 g/cm^3^. At the inception of the impregnation process, the C/C composites displayed low density, and the material structure contained numerous large pores, facilitating the mesophase pitch to permeate through these cavities. With the ensuing density enhancement of the material, the proportion of macropores decreased. Despite the mesophase pitch having a high carbon residue, its elevated softening point, viscosity, and molecular weight resulted in a suboptimal impregnation effect on the micropores. To further augment the performance of the C/C composite, medium-temperature coal pitch, characterized by its low softening point and low average molecular weight, was chosen as the impregnating agent to densify the C/C composite. As evidenced in the figure, the densification rate was high at the initial impregnation stage. With the rise in the number of impregnation cycles, the densification rate of the C/C composite started to plateau. Nonetheless, the medium-temperature coal pitch exhibited a positive impregnation effect on the high-density C/C composites, and the density of the C/C composites achieved 2.02 g/cm^3^ after densifying with medium-temperature coal pitch thrice.

### 3.2. Morphology and Structure of 3D C/C Composites

**Figure 4:**
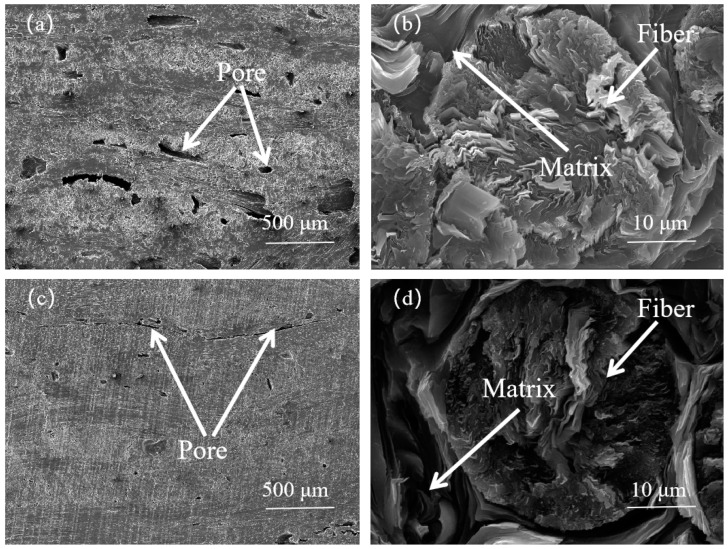
SEM images of 3D C/C composites in the X (Y) direction: (**a**,**b**) C/C-MPI, (**c**,**d**) C/C-MPI-IPI.

Figure 4 displays the SEM images of the cross−section of the 3D C/C composites. The microstructure of the C/C composite remained intact post mesophase pitch impregnation and hot pressing–carbonization. Following multiple rounds of mesophase pitch impregnation, several voids were still present in the C/C-MPI. The mesophase pitch-based carbon fibers formed a graphite sheet with a fold-radiative structure. The mesophase pitch matrix carbon was tightly enfolded around the carbon fibers, and a portion of the matrix carbon was embedded within the split angle of the carbon fibers, wherein the graphite layer was distinctly visible. This observation suggests that the carbon fibers and the mesophase pitch matrix carbon had a strong bond. Furthermore, the voids in the C/C-MPI-IPI were significantly diminished following multiple impregnations with the medium-temperature coal pitch. The graphite sheets that remained in the composite material post carbonization and graphitization exhibited a structure identical to the carbon–graphite sheets of the mesophase pitch matrix, rendering them indistinguishable based on microscopic morphology. The matrix carbon–graphite sheets in the C/C-MPI-IPI were arranged haphazardly with no specific orientation. The pitch-based carbon fibers and PAN-based carbon fibers in the C/C composite were organized in a methodical manner with strong continuity, serving as a rapid thermal diffusion channel during the heat conduction process, thus maximizing the thermal conductivity of the C/C composite.

### 3.3. Polarizing Structure of 3D C/C Composites

**Figure 5:**
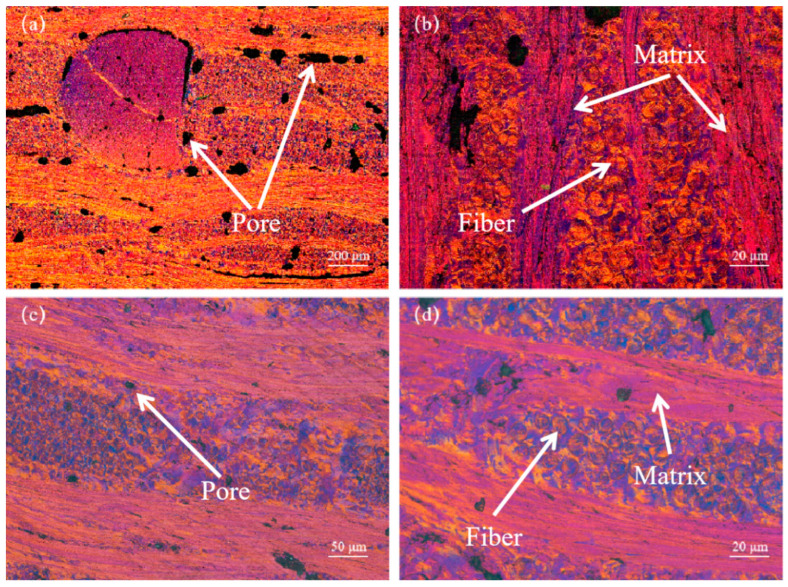
Polarized microscope photo of 3D C/C composites on the X (Y) direction: (**a**,**b**) C/C-MPI, (**c**,**d**) C/C-MPI-IPI.

Figure 5 presents the representative Polarized Light Microscopy (PLM) images of the X and Y planes of the 3D-C/C composites. Evident from the figure, the MPCF exhibiting a large angle split radiative structure are distinctly visible in the C/C-MPI that underwent multiple mesophase pitch impregnations. The mesophase pitch matrix carbon, characterized by excellent orientation and an orderly molecular arrangement, is uniformly dispersed within the primary domain of the carbon composite in a linear pattern. Furthermore, the matrix carbon is tightly enfolded around the carbon fibers, demonstrating effective bonding properties with the fibers. However, after repeated mesophase pitch impregnations, a substantial number of elongated and narrow voids, each less than 50 µm, persist in the C/C-MPI. Following multiple impregnations with medium-temperature coal pitch, the residual matrix carbon in the composite can infiltrate the pores, thereby significantly reducing the voids in the C/C-MPI-IPI. Furthermore, after medium-temperature coal pitch impregnation, the C/C-MPI-IPI displays an isotropic texture with fine flow lines of varying sizes. This texture primarily occurs because the medium-temperature coal pitch, after undergoing rapid carbonization and graphitization heat treatment, does not allow for a complete orientation of the pitch molecules, resulting in the matrix carbon reflecting an isotropic texture in the material structure. This texture may impede heat conduction and impact the thermal conductivity of the C/C composites.

### 3.4. Raman Spectrum Analysis of 3D C/C Composites

**Figure 6:**
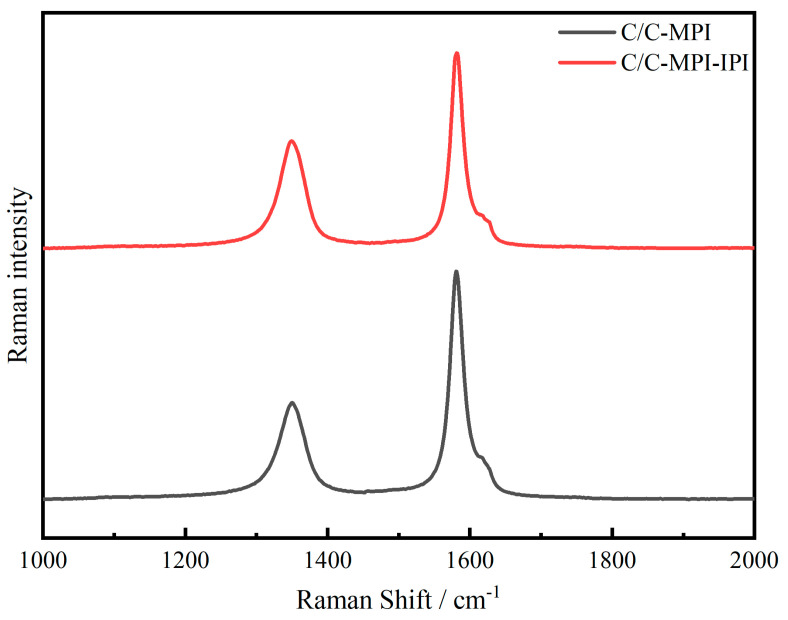
Raman spectra of carbon matrix in 3D C/C composites.

To investigate the impact of matrix carbon on the crystal structure of C/C composites, micro laser Raman spectroscopy was employed in this segment to characterize the structural parameters of the matrix carbon within the composites. Furthermore, the carbon fibers and matrix carbon were analyzed by selecting micron regions and stripping them apart. Figure 6 presents the Raman spectrum of the matrix carbon in 3D C/C composites, while Table 2 lists the corresponding peak intensities and R values derived from quantitative computation of the Raman spectrum, which represents the strength ratio of peak D and peak G (ID/IG) and indicates the defect in the graphite material. As can be observed from the figure, C/C-MPI exhibits a more pronounced D-peak intensity and a less significant G-peak intensity.

As per the values in the table, the R values for C/C-MPI and C/C-MPI-IPI are 0.24 and 0.52, respectively. A smaller R values indicates less defects in graphite materials. These results suggest a higher content of amorphous carbon in the C/C-MPI-IPI matrix carbon. This outcome is primarily due to the presence of a significant quantity of medium-temperature coal pitch matrix carbon within the C/C-MPI-IPI, which is optically isotropic and characterized by a disorganized molecular arrangement and poor orientation. The elevated carbon content of the mesophase pitch matrix enhances the overall graphitization degree and orientation of the C/C composite.

### 3.5. XRD Analysis of 3D C/C Composites

**Figure 7:**
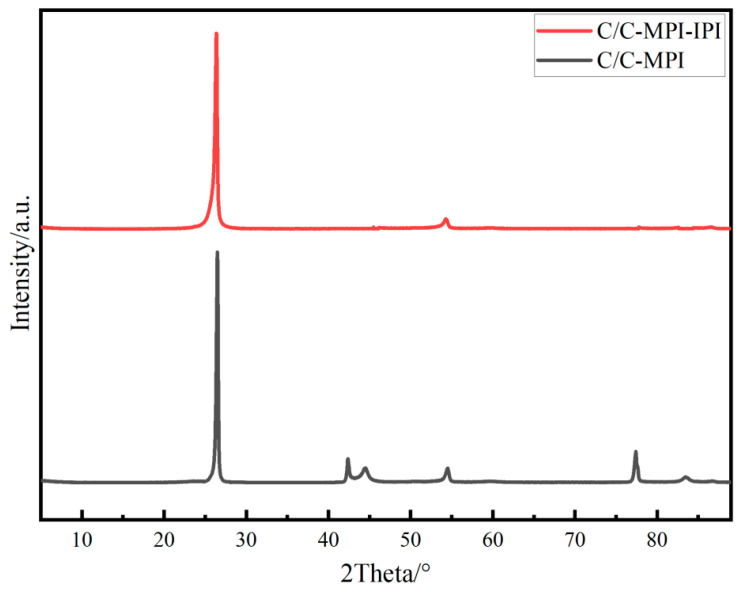
XRD patterns of the X (Y) direction cross section of 3D C/C composites.

Figure 7 illustrates the XRD pattern of the cross-section in the X (Y) direction of the 3D C/C composites. The strongest characteristic peak of graphite is the (002) peak, located at 2θ = 26.5°. With the increase in the degree of graphitization and the size of graphite micro-crystallites, the peak intensity of (002) will gradually increase, and the diffraction peak will be sharper. The diffraction peaks of the (002) and (004) crystal faces in C/C-MPI are manifestly more intense and sharper, indicating superior orientation and greater graphite lamellar stacking height within the C/C composites. Additionally, the intensity of the (110) crystal plane diffraction peak in C/C-MPI is stronger, and the peak shape is more defined, with the appearance of a notably distinct (100) crystal plane diffraction peak. These results imply that the graphite microcrystals in C/C-MPI have formed a clear three-dimensional ordered packing structure, suggesting better crystal development and larger microcrystal size. The variation in the crystal structure of C/C composites at different densification stages can primarily be attributed to differences in the matrix carbon structure. The C/C-MPI matrix carbon contains more graphite carbon, and a substantial amount of disordered carbon is present in the C/C-MPI-IPI matrix carbon. As a result, the (002) characteristic peak intensity of C/C-MPI-IPI is attenuated, and the peak shape is broadened.

### 3.6. Thermal Conductivity of 3D C/C Composites

For C/C composites, the thermal conductivity at room temperature is chiefly determined by the mean free path of phonons. Table 3 exhibits the thermal diffusivity and thermal conductivity of 3D C/C composites. As can be observed, the thermal conductivity of the C/C composites has been notably improved compared with the C/C composites prepared by impregnating and densifying with CVI and furan resin [20]. This improvement can mainly be attributed to the superior regularity of the microcrystalline arrangement of carbon graphite through the pitch matrix, as well as the larger microcrystalline size and mean free path of phonons, leading to improved thermal conductivity. The C/C-MPI has a density of 1.83 g/cm^3^ and a thermal diffusion coefficient of 261 mm^2^/s along the X and Y directions. The axial thermal diffusivity of C/C-MPI-IPI sees a slight increase after the introduction of medium-temperature coal pitch matrix carbon, which could be due to the poor orientation of carbon graphite microcrystals and the disorder in molecular arrangement. However, the gaps within the C/C composite are entirely filled with pitch carbon, reducing the collisions between phonons and material defects and resulting in less energy scattering. This scenario enhances the mean free path of phonons to some extent, causing a minor increase in the thermal conductivity of the C/C composite. It is possible to conclude that the matrix carbon and material structure significantly influence the thermal conductivity of C/C composites.

### 3.7. Thermal Conductivity Analysis of C/C Composites

The fundamental structure of the reinforcement and matrix of the carbon composite is the graphite structure. The thermal conductivity of graphite materials is not only related to its bulk density [21], porosity [22], etc. but also closely related to its microstructure (microcrystalline size and crystal orientation). The high thermal conductivity of graphite materials mainly comes from the firm covalent bond between carbon atoms and the highly ordered lattice arrangement, and heat transfer mainly depends on the non-harmonic vibration of the elastic lattice (interaction of phonons) [23]. The thermal conductivity can be expressed by the Debye formula: λ = 1/3 C·V·L. C is the heat capacity per unit volume, V is the propagation speed of phonons, and L is the mean free path of phonons. For graphite materials with a relatively complete graphite structure, the heat conduction rate at room temperature is mainly determined by the mean free path L of phonons, and L is related to the plane size La of graphite microcrystals. The exceptional thermal conductivity of the 3D C/C composites prepared in this study can be attributed to the following factors. (1) The process of mesophase pitch impregnation and hot pressing–carbonization not only significantly elevated the fiber volume fraction of the C/C composite but also facilitated further orientation of the mesophase pitch within the composite material’s pores. Following carbonization and graphitization, the matrix carbon–graphite laminates were well defined, and the laminate direction was parallel to the fiber axis’s elevation. (2) Post low-temperature carbonization treatment, the surface of the mesophase pitch-based carbon fibers retained a substantial number of active functional groups, fostering improved bonding with the carbon matrix. The matrix carbon graphite sheet and carbon fiber graphite sheet interweave, as evident from the microscopic structure diagram. (3) The density of the carbon composite reached 2.02 g/cm^3^ following several densification processes, and the further rectification of defects, such as pores within the composite, helped minimize phonon energy scattering.

## 4. Conclusions

Highly thermally conductive carbon/carbon (C/C) composites were synthesized from a 3D preform. These were orthogonally woven using low-temperature carbonization mesophase pitch-based fibers and commercial PAN-based carbon fibers and were densified through a process of hot pressing–carbonization and multiple mesophase pitch impregnations (MPI), followed by coal pitch high-pressure impregnation (IPI). The key conclusions drawn from the study are as follows: Firstly, molding can enhance the orientation of the mesophase pitch within the C/C composite material. Post carbonization and graphitization, the alignment of the matrix carbon–graphite sheet is parallel to the elevation of the fiber axis. Secondly, the viscosity of the medium-temperature coal pitch is low, which leads to effective impregnation in high-density C/C composite materials. Such impregnation can decrease the presence of internal pores and defects within the composite materials, thereby improving the material’s thermal conductivity. Thirdly, the matrix carbon significantly impacts the crystal structure and thermal conductivity of C/C composites. The high degree of graphitization of the mesophase pitch augments the microcrystalline size of the C/C composites and improves their thermal conductivity. Conversely, the orientation of isotropic pitch carbon is not high and contributes minimally to the thermal conductivity of C/C composites. Fourthly, the density of the C/C composite was found to be 1.83 and 2.02 g/cm^3^, and the thermal conductivity in the X (Y) direction was measured at 358 and 400 W/(m·K), respectively, following multiple impregnations with mesophase pitch and subsequent impregnation with the medium-temperature coal pitch.

## Figures and Tables

**Table 1:** Physical properties of reinforced fibers in C/C composites (with MPCF treatment temperature at 3000 °C).

Type	The Tensile Strength/MPa	Young’s Modulus/GPa	The Diameter/μm	Thermal Conductivity/W/m·K
MPCF	2520	862	12~16	735
PAN-CF	4200	240	7	47

**Table 2:** D-peak, G-peak strength, and R value of matrix carbon in 3D C/C composites.

Sample	D	G	R
C/C-MPI	1304	3056	0.426
C/C-MPI-IPI	1446	2619	0.552

**Table 3:** Thermal diffusivity and thermal conductivity of 3D C/C composites in X (Y) direction.

Sample	Density/g/cm^3^	TD/mm^2^/s	TC/W/(m·K)
C/C-MPI	1.83	261	358
C/C-MPI-IPI	2.02	264	400

## Data Availability

Data are contained within the article.
